# “Redirecting an anti-IL-1β antibody to bind a new, unrelated and computationally predicted epitope on hIL-17A”

**DOI:** 10.1038/s42003-023-05369-x

**Published:** 2023-09-29

**Authors:** Sharon Fischman, Itay Levin, Jean-Michel Rondeau, Marek Štrajbl, Sylvie Lehmann, Thomas Huber, Guy Nimrod, Régis Cebe, Dotan Omer, Jiri Kovarik, Shmuel Bernstein, Yehezkel Sasson, Alik Demishtein, Tomer Shlamkovich, Olga Bluvshtein, Noam Grossman, Reut Barak-Fuchs, Michael Zhenin, Yair Fastman, Shir Twito, Tal Vana, Nevet Zur, Yanay Ofran

**Affiliations:** 1 Biolojic Design LTD, Rehovot, Israel; 2 Novartis Institutes for Biomedical Research, Basel, Switzerland; 3 The Goodman Faculty of Life Sciences, Nanotechnology Building, Bar Ilan University, Ramat Gan, Israel; 4 Present Address: Enzymit LTD, Ness Ziona, Israel; 5 Present Address: Ridgelinediscovery, Basel, Switzerland; 6 Present Address: EmendoBio Inc., Rehovot, Israel; 7 Present Address: Anima Biotech, Ramat-Gan, Israel

**Keywords:** Biologics, X-ray crystallography, Protein design

## Abstract

Antibody engineering technology is at the forefront of therapeutic antibody development. The primary goal for engineering a therapeutic antibody is the generation of an antibody with a desired specificity, affinity, function, and developability profile. Mature antibodies are considered antigen specific, which may preclude their use as a starting point for antibody engineering. Here, we explore the plasticity of mature antibodies by engineering novel specificity and function to a pre-selected antibody template. Using a small, focused library, we engineered AAL160, an anti-IL-1β antibody, to bind the unrelated antigen IL-17A, with the introduction of seven mutations. The final redesigned antibody, 11.003, retains favorable biophysical properties, binds IL-17A with sub-nanomolar affinity, inhibits IL-17A binding to its cognate receptor and is functional in a cell-based assay. The epitope of the engineered antibody can be computationally predicted based on the sequence of the template antibody, as is confirmed by the crystal structure of the 11.003/IL-17A complex. The structures of the 11.003/IL-17A and the AAL160/IL-1β complexes highlight the contribution of germline residues to the paratopes of both the template and re-designed antibody. This case study suggests that the inherent plasticity of antibodies allows for re-engineering of mature antibodies to new targets, while maintaining desirable developability profiles.

## Abstract (web summary)

A proof of principle approach redirects an anti-IL-1b antibody to bind the otherwise unrelated antigen, IL-17A, highlighting the plasticity of antibody scaffolds that could be manipulated for alternative binding or function.

## Introduction

The antibody framework can accommodate diverse binding sites that can bind virtually any epitope. The number of functional germline antibodies in the serum and mucosa of an individual is much smaller than the immense number of epitopes they can functionally address^1^. A germline antibody is thought to have the potential to bind to multiple different epitopes^2^, a potential that is later reduced in the process of affinity maturation, where the antibody increases its specificity to one epitope. It has been suggested that germline antibodies can bind an antigen through one of many preexisting CDRs conformations^3^. This conformational flexibility of the CDR loops contributes to the positioning of these loops in a shape complementary manner to several targets, enabling utilization of the same CDR residues for binding to several different epitopes.

While undergoing somatic hypermutation, antibodies often become more rigid^2,4^, increasing their affinity to one epitope at the cost of the loss of the ability to bind other epitopes. Somatic hypermutations, according to this observation, may favor one of the multiple possible conformations, and improve affinity and specificity toward one target epitope from amongst multiple possible ones. It has been shown that even in mature antibodies most of the energy of binding of the antigen comes from amino acid positions that did not undergo somatic hypermutation and maintain their germline residues^5^.

Mature antibodies can still maintain some polyspecificity^3,6^. For example, the SPE7 antibody specifically binds several different haptens in addition to a structurally different cyclic peptide. Interestingly SPE7 has 96.9% and 98% identity in its heavy and light chains, respectively, to their closest germline V genes. This phenomenon is found as well with the therapeutic antibody Bebtelovimab, which binds different strains of SARS-Cov-2^7^, and has high sequence identity to germline genes (H and L chains with 94% and 95% identity, respectively).

Specific antibody frameworks are commonly used as templates for large libraries for antibody engineering. For example, Trastuzumab (Herceptin) is often used as a library template for discovering new clinical candidates, because of its stability and good in-human PK/PD profile. However, in most cases, the sequence of the antibodies emerging from these libraries is quite different from the sequence of the template antibody^8,9^. Indeed, in the PDB^10^ there are quite a few examples of antibodies that are based on the Trastuzumab framework and bind unrelated antigens^9,11^. In addition, it has been shown that a library of Trastuzumab-based variants with mutations in the L chain CDRs enabled the engineering of Trastuzumab variants that in addition to Her2, bind novel targets. In this study the template was selected based on structural analysis of the Trastuzumab-Her2 complex, and variation (either full randomization or variation that mimics the natural diversity) was introduced at solvent-exposed L chain CDR positions, resulting in a large library of ~10^10^ variants. In general, the utilization of large general libraries with broad variation may generate binders with a loss of the original developability profile of the template antigen and require additional engineering and optimization.

In this study, we explore the plasticity of mature antibodies. In contrast to earlier work using a large general library that includes full randomization of several positions^11^, we demonstrate that using a small, focused library, it is possible to re-direct, or re-epitope, a mature antibody to specifically bind to a new antigen that has no sequence or structure similarity to its native antigen, with the introduction of very few mutations to the original template. As a case study, we selected an anti-IL-1β antibody with good biophysical properties as the basis for our design, and with the introduction of only 7 mutations, engineered a novel specificity for this antibody, specifically, binding to human IL-17A. We attempted to keep as much of the original antibody intact to maintain a developability profile similar to the parent antibody. Mutated positions were selected based on structural analysis, to allow for the desired new specificity with minimal effect on antibody properties. Using this approach, we were able to engineer multiple variants that bound human IL-17A with high affinity. We solved the crystal structure of one of the antibodies, 11.003, in complex with the new antigen, human IL-17A. Interestingly, 15/22 of the positions in the anti-IL-1β antibody that were in contact with the original antigen (distance cutoff of 5 Å) are in the interface of the 11.003/IL-17A complex. In addition,11/13 contact residues in 11.003, from CDRs L1, H1, and H2, are residues that are identical in both antibodies. Moreover, all but one of these 11 residues are germline residues. The antibodies presented here, engineered by structure-based design, are biologically functional, and specific, and bind hIL-17A with high affinity while retaining desirable biophysical properties of the original antibody template. Finally, we demonstrate that computational predictions of the epitope, based on the sequence and structure of the original template antibody, AAL160, are consistent with the crystal structure of the 11.003/IL-17A complex, indicating the utility of such methods for antibody engineering.

## Results

The goal of this study was to introduce specificity to IL-17A into a given antibody that does not bind this antigen. To preserve the structural integrity and developability profile of the template antibody, we aimed to accomplish this with as few changes to the sequence of the antibody as possible.

The selected template antibody was AAL160, an anti-IL1β antibody that was isolated from immunized mice^12^. AAL160 has good developability properties and binds to IL-1β with an affinity of 0.4 nM^12^. To introduce IL-17A specificity into AAL160, we employed a structure-guided library design approach, aiming to minimally change the sequence of this specific antibody, to re-epitope it to bind IL-17A.

Independent of the design of these libraries, we also used two machine learning algorithms to predict putative new epitopes for AAL160 on IL-17A. These two algorithms were originally developed to screen possible template antibodies for re-epitoping. However, in this instance, the template was selected in advance, and we only used these algorithms to evaluate the potential complementarity between AAL160 and different surfaces on IL-17A.

### Design of AAL160 libraries

**Fig. 1:**
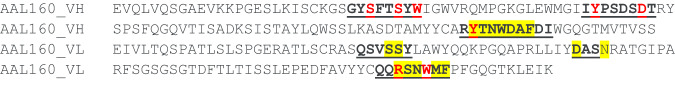
Library A_11.1. AAL160 positions that form specific interactions with IL-1β are shown in red and positions that were varied in the library are highlighted in yellow. CDRs are underlined.

A structure-based library design approach was implemented to introduce IL-17A specificity into the AAL160 antibody, based on the analysis of the interactions of a known anti-IL-17A antibody (h142^13^). The structures of AAL160 and h142 were aligned to identify key residue positions that contribute to IL-17A binding in h142 and are not expected to disrupt the structural integrity upon mutation in AAL160 (Supplementary Fig. 1). 16 positions in CDRs L1, L2, L3, and H3 were chosen for mutation (library A_11.1, Fig. 1) with very limited variation, allowing for either the original residue or the residue in the corresponding position in h142. In addition, an option for the insertion of one amino acid in H3 was included. This design is a highly focused library of only 131,072 sequence variants.

### Computational prediction of potential novel epitopes for engineering the template antibody

We used two computational classifiers to assess the engineering potential of the pre-selected antibody AAL160 to bind new specific epitopes in IL-17A. These predictions were used solely to assess the likelihood of a potential novel IL-17A epitope when using AAL160 as a starting point. The first method utilizes a random forest^14^ classifier we previously described in detail^13^. Briefly, the classifier (referred to as PpRF) takes as input the sequence of an antibody and a 3D structure/model of an antigen. All possible pairwise interactions between an amino acid position in the antibody and an amino acid position in the antigen are computationally screened and assigned a prediction score that corresponds to the likelihood of this amino acid pair interacting within an antibody-antigen interface.

In order to find potential pairwise interactions between AAL160 and IL-17A we used the amino sequence of AAL160 and the 3D structure of IL-17A from the previously published PDB entry 5N7W. The classifier examined all potential pairwise interactions between the antibody and the antigen and the highest score was given to an interaction between GLN27 of CDR L1 in AAL160, and LEU74 in IL-17A.

In order to identify potential epitopes in IL-17A that can be targeted using AAL160 as a template for engineering, we screened the structures of AAL160 and approximately 2500 redundant antibody X-ray structures by docking them into IL-17A using the HexRF method. The docked pose of the AAL160 antibody (Fv structure) with a highly significant HexRF score (ranked 4th in the database screening) suggested a target epitope that was further supported by the PpRF predicted interaction between GLN27 of CDR L1 and LEU74 in IL-17A described above.

The epitope predicted by both PpRF and HexRF classifiers is expected to be biologically functional, as it overlaps with the IL-17RA receptor binding site.

### First-generation selection

To identify AAL160-derived IL-17A binders, the library was screened for IL-17A binding using Yeast Surface Display. Library A_11.1 was subjected to three rounds of FACS sorting. We sorted the top 1% of yeast that expressed the scFv well (FITC—X axis) and were the best binders of IL-17A (APC—Y axis).

While AAL160 displayed no apparent binding to IL-17A in a yeast scFv display setting (Supplementary Fig. 2), library A_11.1 exhibited progress with the percentage of yeast clones positive for both expression and binding increasing from round to round of selection, and clones from this library were isolated and sequenced. Sequencing of several clones revealed a convergence of 5 sequences at the DNA level that translate to 2 sequences at the amino acid level, indicating convergence due to high selective pressure. The clones showed concentration-dependent binding to IL-17A but not to IL-1β, in a FACS analysis, in contrast to AAL160 which binds IL-1β tightly but does not bind IL-17A in this setting (Supplementary Figs. 2 and 3), suggesting that under these conditions, a focused library of ~130,000 variants is sufficient to repurpose the anti-IL-1β AAL160 antibody into an IL-17A binder.

**Fig. 2:**
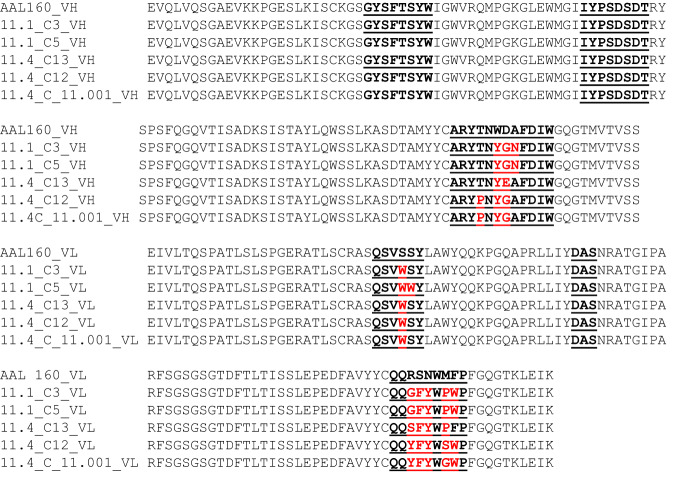
VH and VL sequences of IL-17A-binding clones. Multiple sequence alignment of 11.1_C3, 11.1_C5, 11.4_C13, 11.4_C12, 11.4C_11.001, and AAL160, scFv in heavy to light-chain orientation, CDRs are underlined, mutated residues are marked in red.

As can be seen in Fig. 2, these clones differ from AAL160 by only 9 or 10 residues. In clone 11.1_C3 eight out of nine mutations are in H3 and L3 and one, S164W, is in L1. Interestingly, of the nine mutations, CDR H3 W101Y, (W112Y IMGT numbering) and CDR L3 F228W, (F116W IMGT numbering) are conservative, suggesting that specific binding to IL-17 might be achieved with even fewer mutations. Of note, even though the library A_11.1 design took into account key interactions that are formed between h142 and IL-17A, in a manner analogous to hotspot grafting, several of these mutations are absent in binders that emerged, suggesting that the binders may bind a different epitope than h142, which we now know to indeed be the case (shown below).

### Affinity maturation library

An affinity maturation library was designed based on clones 11.1_C3 and 11.1_C5. This library (library C_11.4) was focused on varying four H3 positions and six L3 positions. Some of these positions had been varied in library 11.1 with a limited diversity and the diversity at these positions was expanded in this library. For example, two positions that were mutated in the first-generation library using binary variation, now were explored using codon NNS encoding for all amino acids. Other positions had varying degrees of variation. Library_C_11.4 had ~700,000 variants.

Library_C_11.4 was screened for clones with both improved IL-17A affinity and thermostability. A high correlation between surface display and thermal stability has been previously demonstrated^15^; we used a slightly modified screening approach in which the library is screened for improved binders while the temperature is elevated from 37°C in the first round to 40 °C in the following rounds. As can be seen in Supplementary Fig. 4, the library was gradually enriched for high-affinity binders that also expressed well as a yeast surface displayed scFv at elevated temperatures, indicating that tight binders that are likely more stable were selected. Additionally, another clone that was sorted under less restrictive conditions was isolated.

Sequencing of the enriched clones revealed that the library converged to two antibody variants. These affinity-matured antibodies showed specific binding to IL-17A by yeast surface display. As can be seen in Fig. 2, clone 11.4_C13 has only 7 mutations relative to AAL160; interestingly, while clone 11.4C_11.001 and clone 11.4_C12, which were selected under high-temperature pressure, resulted in a T100P mutation in CDR H3, clones 11.1_C3 and 11.1_C5 from the first-generation and clone 11.4_C13 from the affinity maturation library which were both selected under more permissive conditions, do not have this mutation. Proline mutations in CDRs are associated with rigidification of CDRs^16^, suggesting that this mutation may have rigidified H3 contributing to the thermostability, and potentially enhancing affinity by reducing entropic penalty of these variants. As such, one of these clones, 11.4C_11.001 was selected for further development and analysis.

### Production and biochemical analysis of BDG11.001

We tested the properties of clone 11.4C_11.001 as a soluble IgG (BDG11.001). We reformatted the scFv and expressed it as an IgG1 as described in the Methods section. SEC analysis indicated that BGD11.001 was homogeneous with more than 95% of the protein migrating in a non-aggregated form; analysis of the peak by SDS PAGE revealed intact IgG. However, while a typical antibody elutes at ~13.5 ml on a Superdex 200 10/300 column, BDG11.001 eluted at 16.2 ml indicating a possible interaction with the column (Supplementary Fig. 5).

**Fig. 3:**
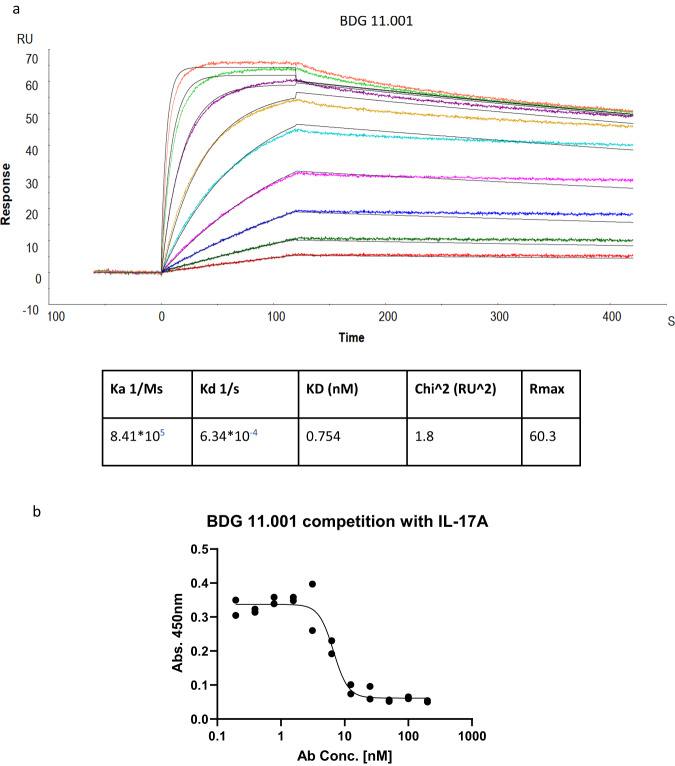
BDG11.001 binds IL-17A and competes with IL-17RA. **a** SPR binding kinetics of BDG11.001 to human IL-17A. **b** BDG11.001 competition with the IL-17RA receptor. hIL-17A was coated on the ELISA plate well, and BDG11.001, at a concentration range of 0–200 nM, competed with IL-17RA by subsequent addition of the receptor for a short period of time. Absorbance at 450 nm correlates with IL-17 RA binding to IL-17A. Confidence intervals of IC50 for BDG11.001 is 5.4–9. Analysis was done in GraphPad Prism 9 using [Inhibitor] vs. response—variable slope (four parameters). Data are *n* = 2 technical replicates.

The antibody affinity for human IL-17A was tested by SPR as described in the Methods section. As can be seen in Fig. 3a, BDG11.001 binds IL-17A tightly with a sub-nanomolar affinity of 0.75 nM.

To determine if BDG11.001 has a comparable melting point to AAL160, both antibodies were tested by DSF using sypro orange fluorescence temperature shift^17^. Tm1 was determined to be 65°C for both antibodies and Tm2 was 76 °C and 79 °C for BDG11.001 and AAL160, respectively, indicating that BDG11.001 has similar thermostability characteristics as the template antibody AAL160 (Supplementary Fig. 6).

PpRF and HexRF both predicted an epitope on IL-17A whose binding would result in blocking of the IL-17RA receptor. To test if the antibody is binding a functional epitope BDG11.001 was subjected to an ELISA competition assay with recombinant IL-17A receptor (IL-17RA) as described in the methods section. As can be seen in Fig. 3b, BDG11.001 competes with the IL-17RA receptor in a dose-dependent manner, suggesting that as also predicted for the AAL160 template, BDG11.001 binds an epitope that results in blocking the IL-17A receptor binding.

**Fig. 4:**
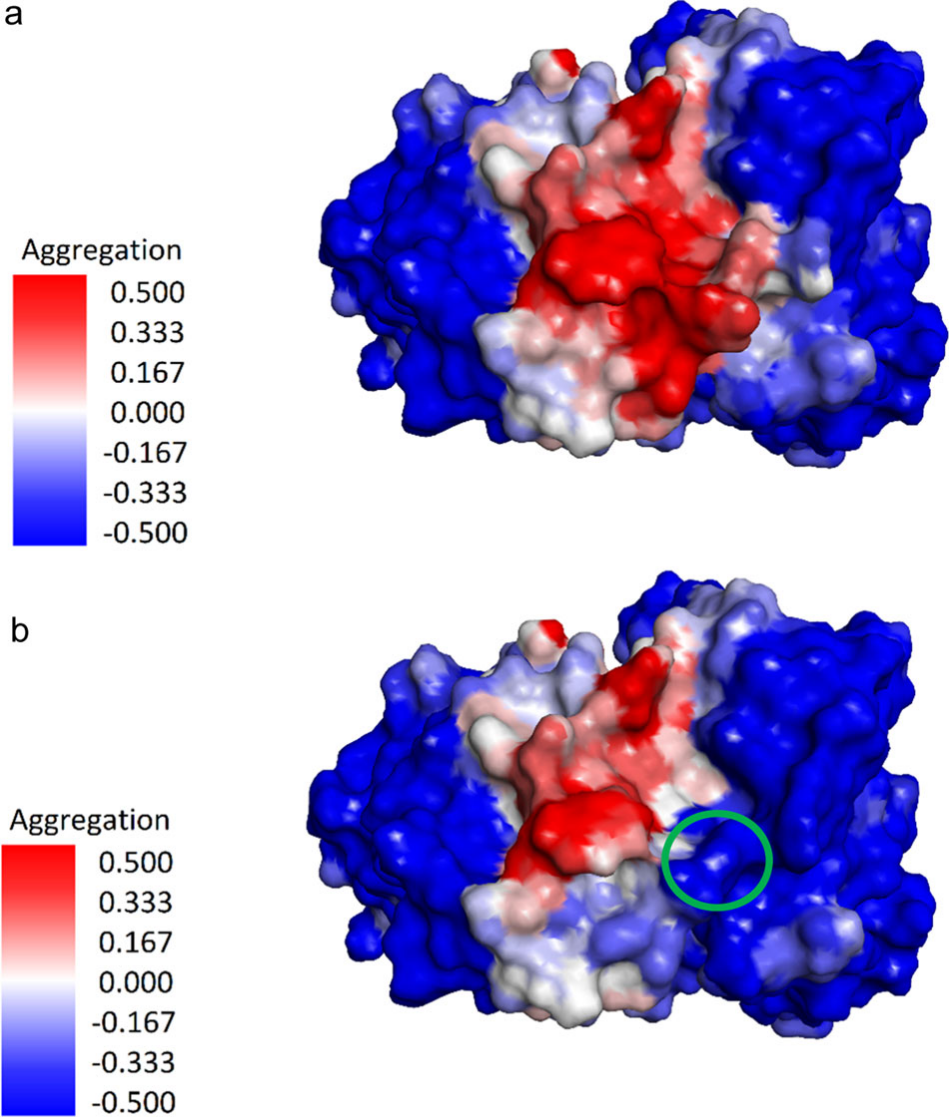
Aggregation propensity of models. **a** Model of clone 11.4_C12. **b** Model of a triple mutant of this clone (L_Y93N, L_W94N, L_S95G) showing an improved aggregation propensity prediction. The surfaces of the models are color-coded according to the calculated aggregation propensity, based on an implementation of the SAP method, with red indicating the aggregation-prone regions. A green circle marks the area of the mutations.

BDG11.001 expressed well and eluted from a Superdex 200 SEC column with no sign of a major aggregation peak, however it showed a 20% longer retention time, vs the 13.5 ml retention time exhibited by AAL160, which is typical for an IgG. To improve the biophysical properties of BDG11.001 we identified residues that may contribute to non-specific interactions with the SEC column. We hypothesized that these residues may be identified by the prediction of aggregation propensity, calculated on a model of a clone emerging from the affinity maturation library C_11.4 (clone 11.4_C12). This clone was modeled, with the homology modeling tools in the Schrödinger software^18^, using AAL160 as a template. The model was analyzed using the spatial aggregation propensity method (SAP)^19^ as implemented in Discovery Studio on a single structure and with the AggScore tool from Schrödinger^18^. Positions L_Y93 (Y109 IMGT) and L_W_94 (W114 IMGT) had the highest aggregation scores with the AggScore tool. In addition, these residues are in the region that showed high aggregation propensity with the Discovery Studio SAP prediction and are also present in BDG11.001. Therefore, in order to generate clones with improved biophysical characteristics L_Y93 and L_W94, as well as L_W30 (W36 by IMGT) which is also part of the surface predicted to be aggregate prone by both methods, were selected for mutation to polar residues. (Fig. 4).

### Generation and characterization of improved variants

Using these aggregation propensity predictions, we generated five mutants; four mutants had a single substitution of Y93N, W94N, W30S, W30F, and one had a double mutation of Y93N and W94N. A list of all variants from this paper is in Supplementary Fig. 7.

While BDG11.008 (Y93N, W94N) expressed as a soluble antibody, it precipitated at 4°C 72 h post purification and was not analyzed; all the other mutants expressed well, with less than 5% aggregates after protein A purification, as apparent from analytical SEC analysis. Additionally, compared to BDG11.001, their SEC retention time was reduced, leaning closer to the typical IgG retention of 13.5 ml (Supplementary Fig. 8).

**Fig. 5:**
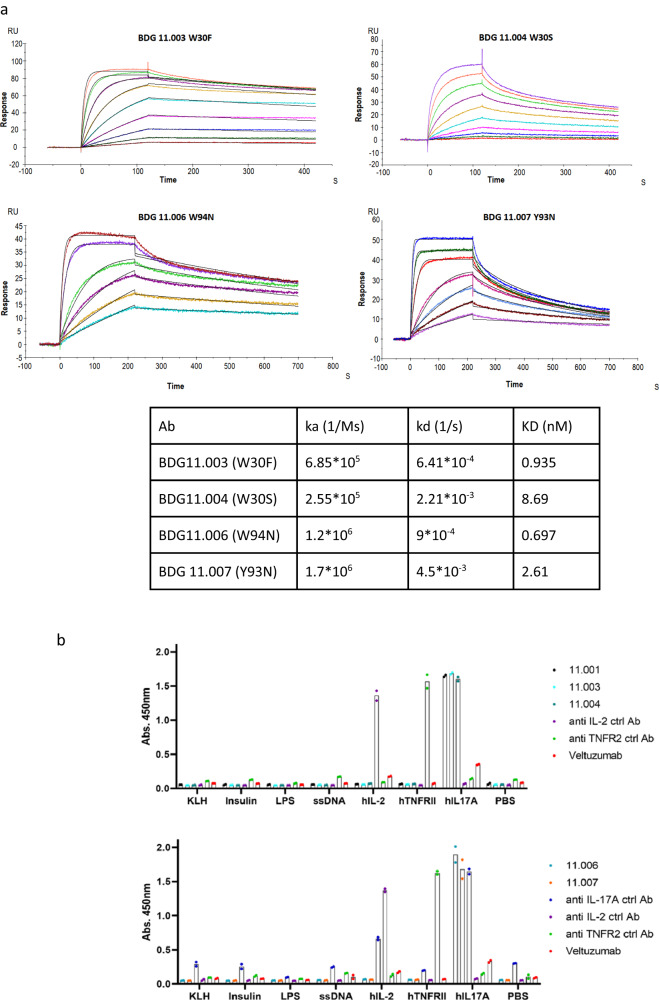
Anti IL-17A antibodies bind IL-17A with high specificity. **a** SPR binding kinetics of BDG11.003, BDG 11.004, BDG, 11.006 and BDG11.007 to human IL-17A. **b**. Polyspecificity ELISA binding assay of BDG11.001, BDG11.003, BDG11.004 (upper panel), BDG11.006 and BDG11.007 (lower pane). Anti hIL-17A antibodies show to have specific binding to hIL-17A and no binding to KLH, insulin, LPS, ssDNA, hIL-2, hTNFR2, and BSA. Positive controls for hIL-2, hTNF**R2,** and hIL-17A were anti-IL-2 antibody, anti-TNFR2 antibody, and anti-IL-17A antibody respectively. Analysis was done in GraphPad Prism 9 using 2-way ANOVA. Data are *n* = 2 technical replicates.

The mutants were also analyzed for binding to human IL-17A by SPR and cross-blocking of the IL-17RA receptor. As can be seen in Fig. 5a and Supplementary Fig. 9, all the mutants bound IL-17A with a binding constant ranging from 0.7 nM to 8.7 nM (Fig. 5), and all mutants cross-blocked the IL-17RA receptor.

In order to confirm the specificity of the engineered antibodies to the new target antigen, we tested the binding of BDG11.001- BDG11.007 to a panel of protein and biological molecules. As can be seen in Fig. 5b, the re-epitoped antibodies show high specificity to IL-17A. Interestingly, the non-specific interactions of BDG11.001-BDG11.007 are lower than that of reference antibodies, indicating that these AAL160-based re-epitoped antibodies are indeed specific.

We also tested the ability of the re-epitoped antibodies to cross-block the binding of human IL-17A to its cognate receptor in a cell-based, biologically relevant setting. BJ fibroblasts cells naturally express the IL-17RA receptor and upon activation with IL-17A secrete IL-6. To determine the IC50 of the re-epitoped antibodies in this setting, we treated the BJ fibroblasts with IL-17A and various concentrations of the antibodies, then 24 h post treatment measured secreted IL-6 levels.

As can be seen in Table 1 BDG11.001 blocked IL-17A-dependent secretion of IL-6 with a single-digit nM IC50 and 11.003 with a low double-digit nanomolar IC50 while AAL160 did not show significant inhibition of IL-6 production with concentrations of up to 1000 nM. These results indicate that in a biologically relevant cellular setting, binding of BDG 11.001 and 11.003 to IL-17A inhibits the cellular signaling cascade.

**Table 1:** BJ fibroblast IL-17A dependent, IL-6 secretion assay.

mAB	IC50 (nM)
BDG 11.001	4.8 ± 1.9
BDG 11.003	33.9 ± 16.8
BDG 11.004	162.6 ± 92.5
BDG 11.006	50.5 ± 37.1
BDG 11.007	286.5 ± 228
AAL160	>1000

IC50 values for blockade of IL-17A mediated signals are presented (mean ± SD on n-3 individual experiments).

### Structure

**Fig. 6:**
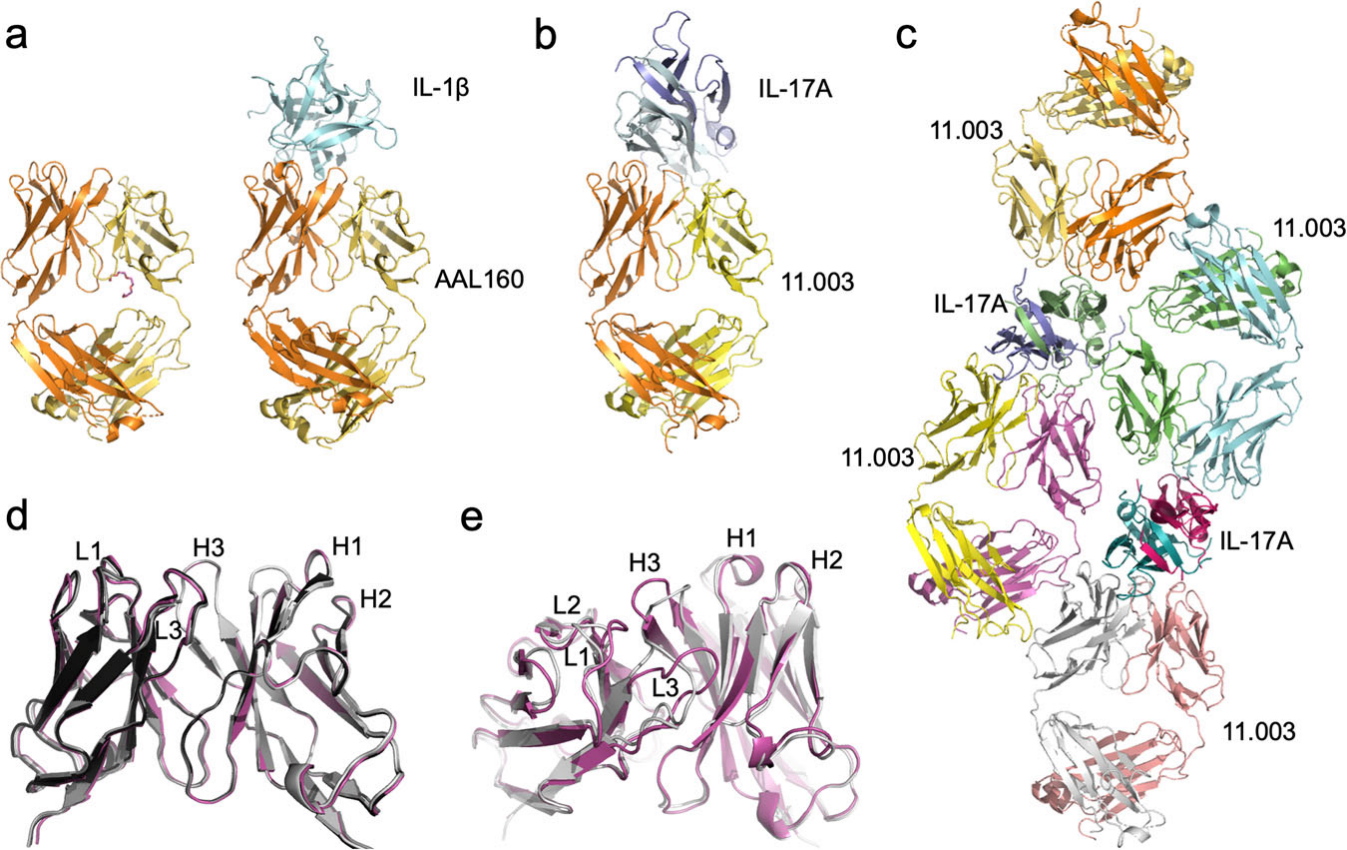
Crystal structures of AAL160, the AAL160/IL-1β complex, and the 11.003/IL-17A complex. **a** Crystal structures of the free AAL160 Fab and of its complex with IL-1β. **b** Structure of the 11.003 Fab complex with IL-17A. **c** Asymmetric unit of the 11.003 Fab/IL-17A crystal. **d** Overlay of the four 11.003 VHVL domains present in the asymmetric unit. **e** Overlay of the VHVL domain of AAL160 as observed in the IL-1b complex (magenta ribbon) and of the VHVL domain of 11.003 (chains H,L; gray ribbon) as observed in the IL-17A complex.

**Fig. 7:**
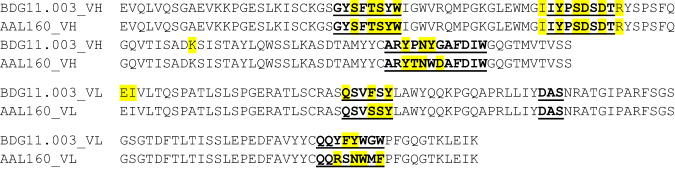
Comparison of AAL160 and BDG11.003 Paratopes. AAL160 paratope and BDG11.003 paratope are highlighted in yellow. Paratopes are calculated as residues within 5Å of the antigen using antigen chains GI and 11.003 chains HL.

To elucidate how relatively small changes to the sequences of AAL160 CDRs resulted in a strong and specific binding to IL-17A, we solved the crystal structure of the 11.003 Fab/IL-17A complex (Table 2). 11.003 was produced in a Fab format, as described in the Methods section. The complex of 11.003 Fab and IL-17A was crystallized and the structure was determined at 1.9 Å resolution. The structures of the parent AAL160 Fab in the free state and in complex with human IL-1β had been determined previously^12^ but are reported here (Fig. 6a and Supplementary Tables 1 and 2). Like all other anti-IL-17A antibodies of known X-ray structure to date, 11.003 binds IL-17A with a stoichiometry of two Fabs per cytokine homodimer. The asymmetric unit of the crystal comprises two full homodimeric complexes (Fig. 6c). While the four binding interfaces are very similar overall, one interface shows additional interactions with IL-17A residues Thr33 to Arg39, which are stabilized by crystal contacts in this case but otherwise disordered in all other IL-17A subunits. We believe that the interactions made by these flexible IL-17A residues are weak overall and only transient in solution. Disregarding their contribution, the binding interface is large, exhibits a high degree of surface complementarity (Supplementary Table 1), and features a large number of water molecules mediating H-bonded interactions. The 11.003 epitope on human IL-17A is mainly located within the first and second β-hairpins of a single IL-17A subunit. The contribution of the second subunit to the epitope is minimal, except in the case of the IL-17A subunit for which the flexible coil residues Thr33 to Arg39 are ordered and stabilized by crystal packing contacts. The 11.003 paratope involves all CDRs except L-CDR2, with the heavy chain contributing approximately 60% of the total buried antibody surface. Structural overlays show that the four antigen-combining sites in the asymmetric unit of the crystal are highly similar, with essentially identical CDR loop conformations (Fig. 6d). In contrast, superimposition with the parent AAL160 antibody reveals significant changes in the conformation of the L1, L3 and H3 CDR loops (Fig. 6e). The conformational change of the L1 loop is particularly worthy of note, as 11.003 and AAL160 differ only by one point mutation in this region (Phe30 to Ser). Interestingly, a detailed comparison^20^ of the AAL160/IL-1β and 11.003/IL-17A complexes reveals that both complexes have similar surface complementarity (0.78 vs 0.72) and the total amount of buried surface (1602 Å^2^ compared to 1700 Å^2^, Supplementary Table 1). Moreover, of the 22 AAL160 CDR positions in contact with IL-1 β, 15 are also utilized in the 11.003 complex, with 13 bearing identical side-chains (Fig. 7). We also note that, among the latter, 10 side-chains correspond to the germline sequence. (Supplementary Table 2).

**Table 2:** X-ray data collection and refinement statistics.

	Free AAL160 Fab	AAL160 Fab/IL-1β complex	11.003 Fab /IL-17A complex
Data collection			
Space group	P2_1_2_1_2_1_	C2	P2_1_2_1_2_1_
Cell dimensions			
*a*, *b*, *c* (Å)	62.17, 89.83, 123.73	185.79, 37.17, 97.07Å	83.996, 107.335, 269.297
*α*, *β*, *γ* (°)	90.000, 90.000, 90.000	90.000, 114.92, 90.000	90.000, 90.000, 90.000
Resolution (Å)	2.00 (2.07–2.00)	3.30 (3.42–3.30)	1.899 (1.931–1.899)*
*R*_merge_	0.050 (0.381)	0.159 (0.505)	0.130 (2.612)
*R*_meas_			0.138 (2.752)
*R*_*pim*_			0.045 (0.856)
*I*/σ*I*	11.7	5.3	11.1 (1.1)
CC^1/2^ (%)			0.998 (0.292)
Completeness (%)	99.9 (100.0)	97.7 (86.1)	98.8 (98.0)
Multiplicity	8.4	4.1	10.2 (10.9)
Refinement			
Resolution (Å)	16.38–2.00	29.34–3.30	134.65–1.899
No. reflections	47,440	9257	188,538
*R*_work_/*R*_free_	0.196/0.217	0.238/0.269	0.2013/0.2273
No. atoms			
Protein	3271	4456	16,443
Water	317	0	1507
Buffer components	98 (7x PEG 200)	0	18 (3 glycerol mol.)
*B*-factors (Å^2^)			
Fab Lc (chain A, C, E, L)	37.4	65.1	35.5, 41.1, 48.9, 37.8
Fab Hc (chain B, D, F, H)	35.8	45.6	36.8, 39.3, 42.3, 37.9
Antigen (chain G, I, J, K)	-	70.9	46.0, 42.8, 48.9, 42.6,
Water (chain W)	47.2	-	46.6
R.m.s. deviations			
Bond lengths (Å)/angles (°)	0.008/1.01	0.008/1.07	0.008/0.98

**Fig. 8:**
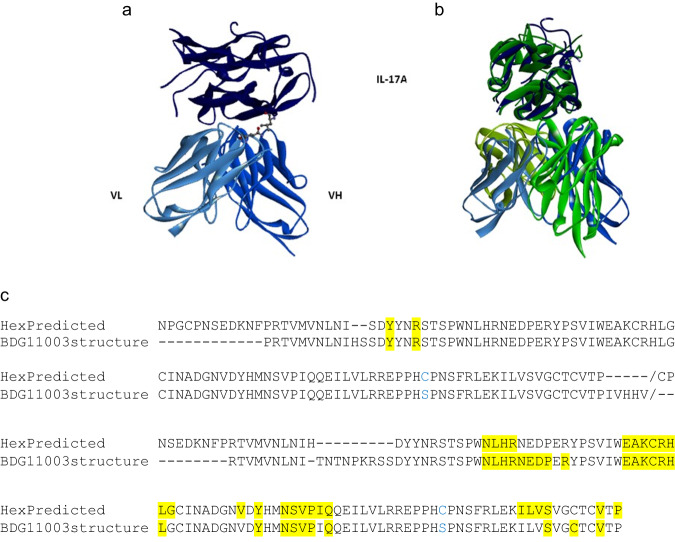
Comparison of the computationally predicted epitope with the 3D structure. **a** PpRF prediction of residue-residue contacts between AAL160 and IL-17A mapped onto the crystal structure of the 11.003/IL-17A complex. **b** HexRF docking pose prediction for AAL160 and IL-17A (green) compared with the crystal structure of 11.003 clone bound to IL-17A (blue). **c** IL-17A epitope/contact residues. IL-17A residues within 5 Å of AAL160 in the HexRF docked pose (upper sequence), and in the 11.003/IL-17A complex crystal structure (lower sequence) are shown in yellow. The IL-17A epitope predicted by HexRF docking of AAL160 and IL-17A is consistent with the epitope observed in the IL-17A-11.003 complex crystal structure.

The computational epitope predictions based on the template antibody, AA160, are consistent with the epitope observed in the crystal structure of the 11.003/IL-17A complex (Fig. 8). Interestingly, as predicted, the crystal structure of 11.003 bound to IL-17A confirms that 11.003 epitope on IL-17A is different than that of h142 (Supplementary Fig. 1). This observation highlights potential limitations of using methods similar to hotspot grafting as a means of epitope targeting, in contrast to success utilizing computational methods for epitope prediction.

## Discussion

Traditional antibody discovery relies on large-scale screening of sequences stochastically generated either in vivo or in vitro^21–23^. Typical campaigns may require screening of up to 10^11^ variants^24,25^ to get good binders that may or may not be functional and developable. Computational design attempts to circumvent this stochastic process by designing sequences that will possess the desired characteristics^26^. Computational methods have been used to generate antibodies with novel epitope-specificity to Keap1, insulin, IL-17A, and ACP2 with affinities ranging from 4 nM to 50 nM^27,28^. Previously, we described a computational approach for antibody re-epitoping, such that they bind a new antigen at a predefined epitope^13^. In that study, a specific antibody template predicted to have the potential for engineering novel antigens and epitope-specificity was selected from a database of existing antibodies and engineered to bind a pre-determined epitope. Here we took this approach a step further. Instead of searching for a new template, we predefined not only the new desired target but also one specific template antibody and showed we could use computational tools to determine a potential target epitope. Our goal was to start from the anti-IL-1β antibody AAL160 and engineer it to bind IL-17A via the introduction of a minimal amount of mutations. We used a focused library designed to maintain the structural integrity of the template and its developability profile. AAL160 is a well-characterized antibody with good physicochemical attributes. A small and highly focused library, consisting of only ∼130,000 variants, each of which is over 92% identical to the FV of AAL160, sufficed to identify several high affinity and high specificity binders to IL-17A. Polyspecific interactions are a concern of antibody discovery, and they are known to occur occasionally during discovery campaigns^29,30^. The binders presented here showed no non-specific binding, with binding background levels comparable to those of a clinically approved antibody. The lead antibody, 11.003, has a sub-nanomolar affinity to IL-17A, representing fivefold better affinity than previously reported, computationally designed novel binders^13,27,28^.

We show that using the sequence or 3D structure of an existing mature antibody, AAL160 in this case, we can predict a potential new epitope on a completely unrelated target antigen, using both PpRF and HexRF classifiers (Fig. 8a). In addition, the structure-guided HexRF classifier predicts an epitope that is in agreement with the PpRF prediction. Notably, both predictions are based on the original sequence and structure of AAL160 that does not bind IL-17A at all, without taking into account mutations that will enable binding to IL-17A. While PpRF and HexRF epitope prediction classifiers are based on different computational approaches, their epitope predictions are consistent with each other as well as with the crystal structure of the 11.003/IL-17A complex. This result demonstrates the strength of these predictive tools and offers insight as to how they may be applied to antibody engineering challenges. Combining these different classifiers can be a powerful approach for designing antibodies to target a specific epitope^13^. This approach may also be adjusted for in-silico screening of sequences in search of biologically active clones that emerge from large, general libraries, thereby enabling the identification of clones predicted to bind an epitope of interest. In addition, further studies can explore the high-throughput application of these methods for computational epitope prediction for a given antibody template for different target antigens.

A comparison of the crystal structures of the 11.003/IL-17A complex and the AAL160/IL-IL1β complex reveals a large degree of conservation between the paratope residues of each antibody. Of the 21 residues in 11.003 that are in the 5 Angstrom interface with IL-17A, 13 are conserved in the AAL160 paratope. Remarkably, only two of these conserved paratope residues are in H3, and of the rest, all but one of the conserved paratope residues are germline residues. Interestingly, large-scale analysis has demonstrated the contribution of germline paratope residues to antigen binding^5^. The ability of identical residues in the two different antibodies to mediate interactions with the different antigens may be attributed to AAL160’s germline likeness. AAL160 binds IL-1β with high specificity and affinity^12^, yet its VH sequence is 99% identical to germline IGHV5-51*0,1 with only one Gly54 to Ser difference, and its KV sequence is 100% identical to germline IGKV3-11*01^31^ (Supplementary Fig. 10). While AAL160 is a mature antibody, as it was generated via immunization and hybridoma technology, and has 0.4 nM affinity for its ligand, its specificity seems to be determined largely by the sequences of H3 and L3, due to the germline nature of the remaining CDRs. A similar mechanism is observed in 11.003. Both antibodies utilize their conserved germline residues as a large part of the paratope.

Interestingly, the structure of the 11.003/IL-17A complex reveals multiple water-mediated interactions in the antibody-antigen interface, including interactions mediated by the germline residues. This observation is consistent with the finding that antibodies utilize water-mediated interactions to compensate for sub-optimal shape complementarity with an antigen^32,33^.

The effect of affinity maturation on antigen specificity and conformational diversity has been an active field of investigation. Germline, or very close to germline, antibodies may bind with low affinity to several structurally unrelated antigens. These antibodies are inherently more flexible than high-affinity antibodies^34^, which are thought to become more rigid^2,4^. The AAL160 template might represent a middle ground between mature and germline antibody: on one hand it is functional and specific, but on the other hand, some of its CDRs are almost identical to the germline sequence. As such, it may retain some germline properties.

This phenomenon is observed in other mature antibodies that have high sequence identity to germline genes. We searched the SAbDab database for germline-like antibodies (>93% sequence identity to V-gene sequence) that have similar sequences but bind different epitopes. Comparison of the paratopes of these antibodies reveals a number of examples of pairs of germline-like antibodies that have sequence similarity to each other and utilize a conserved paratope to bind unrelated antigens. For example, an antibody targeting Clostridium difficile toxin B (PDB 4NP4) is 90.7% identical (Fv region) to an antibody against IL-13 receptor alpha (PDB 4HWB), and the paratope residues of CDRs H1, H2, and L1 are largely conserved between these antibodies. Another example is anti-PCSK9 (PDB 3H42) and anti-RSV F protein (PDB 6APD) antibodies, which are 91.5% identical (Fv regions), and share a conserved paratope in H1, H2, and part of L3 CDRs (Supplementary Fig. 11). These observations suggest that in some cases near-germline antibodies, like AAL160, can bind their target specifically while still retaining inherent germline structural properties, rendering them suitable candidates for engineering new specificity with just a handful of mutations.

The work described here serves as the basis for important advances in antibody engineering. Previous studies using large general-purpose libraries have demonstrated that novel specificity can be obtained via broad variation of L chain CDRs^11^ or H chain CDRs including randomization of H3^9^. We report here that a small and focused library, with a specific selection of positions to be varied and limited variation at these positions, can yield functional binders to a novel target. Furthermore, the results presented here demonstrate the plasticity of antibody templates through the use of conserved germline residues in diverse paratopes, and offer insights into the selection of appropriate antibody templates for successful engineering. Specifically, in addition to the preference of a starting template that maintains good developability properties, germline residue content of the original paratope or CDRs, can be considered even for mature antibodies. Using binding to IL-17A as a case study, we describe an approach for antibody engineering that has the potential to enhance the design of therapeutic antibodies, and that, in contrast to traditional methods, addresses some of the current challenges in this field, such as template selection and minimization of developability liabilities. In addition, we demonstrate that the design of a small, highly focused library succeeds in achieving novel specificity with the introduction of only 7 mutations. The work described here is a single case study, and as such has the limitation that additional examples would be needed for the broad application of this method. However, we believe this approach has the potential to be generally applicable to antibody engineering. Given the lack of similarity between the antigens (IL-1β and IL-17A) and epitopes, and given that the data we used can be generated for other antibodies and antigen targets, this approach can readily be applied to other antibody-antigen pairs.

The ability to introduce a novel function into one pre-selected template, while maintaining a favorable developability profile, has implications for the development of therapeutic entities, such as bi-specific and dual-specific antibodies^35^, where a given antibody may be a required starting point for engineering. The utilization of computational methods to predict the epitope to be targeted by a template antibody allows for engineering specific functions, such as antagonism or agonism, rather than simply a binder. Finally, the observation that germline paratope residues are repurposed to contribute to the binding of different antigens, offers insights into the optimal characteristics of template antibodies and can guide the selection of templates for engineering.

## Methods

### Library generation

Libraries were constructed based on the AAL160 template antibody by overlapping extension PCR with degenerate oligonucleotides. PCR used to introduce diversity was done using Phusion high fidelity DNA polymerase (New England Biolabs USA, Cat: M0530) according to manufacturer instructions in a 3-step reaction (98 °C for 30 sec, 65 °C for 20 sec, 72 °C for 30 sec, 30 cycles). Subsequently, the DNA fragments were gel purified and assembled in equimolar ratios in a 3-step PCR reaction, as above, but in the absence of primers. The assembled scFv library was amplified using forward and reverse primers adding the yeast surface display expression vector homology recombination sequences at the 5′ and 3′ to the scFv library allowing efficient homology recombination into EBY-100 yeast strain.

scFv libraries were constructed with three repeats of flexible G^4^S linkers between the VH and VL.

### Library screen

Yeast-displayed first-generation and affinity maturation scFv libraries were grown in a SD-CAA selective medium and induced for expression with 2% w/v galactose at 30 °C overnight as described in Chao G et al.^36^. The library was incubated with 500 nM to 10 nM biotinylated human IL-17A in PBS 0.1% BSA for 1 h, then washed three times with PBS 0.1% BSA and labeled with fluorescent-labeled antibodies mouse anti-Myc-FITC (Miltenyi Biotec, USA, cat:130-116-485) streptavidin APC (Jackson Immunoresearch, USA, cat 016-130-084). Post labeling the library was sorted on either BD ARIA III or BioRad S3e Fluorescence Activated Cell Sorter for high-affinity binders of human IL-17A. Gating strategy: EBY-100 cells labeled for FITC fluorescence (expression) above null control, which also showed top 1% APC fluorescence (binding) were considered as best binders and gated for collection. Isolated clones from the final sort were sequenced by extraction of plasmid DNA from the yeast clones using a Zymoprep kit (Zymo Research, USA) and the DNA was sequenced.

For the affinity maturation screen, the library was screened in the same fashion, but the yeast was induced at 37 °C to 40 °C and labeled with 1 nM–100 nM biotinylated IL-17A.

### ScFv reformatting and IgG expression

All clones were reformatted to human IgG1. Selected scFv clones were isolated and the scFv coding DNA was extracted from the yeast. The Heavy-Chain FV and Light Chain FV were PCR amplified and cloned into pSF-CMV-HuIgG1_HC (HC plasmid) and pSF-CMV-HuLambda_LC (LC plasmid), respectively (Oxford genetics, Oxford UK) using standard cloning techniques. To express the IgG, Expi-CHO cells were transfected with LC and HC plasmids at a ratio of 2:1 and grown at 32 °C with 8% CO_2_ for 10 days according to the manufacturer’s instructions (Thermo Fisher Scientific, USA). Subsequently, the cells were harvested, IgGs were purified from the supernatant using protein A beads (Tosoh Bioscience GmbH, Germany) and the buffer was exchanged for PBS.

### Size exclusion chromatography (SEC)

100 μg IgG samples were loaded on a Superdex 200 10/300 column on Akta Explorer (GE Healthcare, USA) at a flow rate of 0.8 ml/min using PBS as the mobile phase. Monitoring of antibody retention time was done at 280 nm.

### SPR

Affinity was determined by SPR analysis on Biacore T100 using a CM5 chip (GE Healthcare, USA) The chip was crosslinked with primary capture antibody against human IgG (Cat: br-1008-39, GE Healthcare, USA) to a target of 8000RU. After cross-linking of the primary antibody, tested antibodies were immobilized on the primary antibody to a target of approximately additional 500RU, and IL-17A analyte in PBS 0.05% tween buffer was injected in a series of two- or three-fold dilutions for each cycle in multi-cycle kinetics strategy. At the end of each cycle the IL-17A analyte and tested antibody were stripped from the chip using 3 M MgCl_2_ and the new tested antibody was loaded again on the chip as described above.

### DSF

12.5 μl of 300 μg/ml tested antibodies were mixed with 12.5 μl of Sypro Orange (S6650 Thermo Fisher, USA) and loaded on a Bio-Rad Light cycler (Bio-Rad, USA), fluorescence (excitation, 515–535 nm; detection, 560–580 nm) was measured from 20 °C to 95 °C in 1°C/60 sec steps, the inflection point was determined by plotting the fluorescence derivative dFL/dTm.

### Polyspecificity assay

High-binding ELISA plates (Greiner Bio-One, Germany) were coated with 50 μl of 5 μg/ml KLH, 5 μg/ml insulin, 10 μg/ml LPS, 1 μg/ml ssDNA, 5 μg/ml human IL-2, 5 μg/ml human IL-17A, 5 μg/ml hTNFR2. Wells were blocked with PBS 0.5% BSA and washed three times with 300 µl PBS-T. Then 50 µl of 100 nM (0.75 μg/well) of indicated antibodies were added to the plate and the plate was incubated for 1 h at RT. Subsequently, the plate was washed three times with 300 µl PBS-T and 50 µl of goat anti-human Fc-HRP conjugated antibody (Jackson, 109-035-008) diluted 1:20,000 in PBS-T was added for an incubation of 20 min at RT. Finally, the plate was washed three times with 300 µl PBS-T. The reaction was developed with 50 μl tetramethylbenzidine (TMB) reagent (Southern Biotech, USA) and stopped with 50 μl 0.5 N H_2_SO_4_. Detection was done on a Synergy LX BioTek (BioTek, USA) plate reader by reading absorbance signals at 450 nM.

### IL-17RA competition with recombinant IL-17A

50 µl of 5 nM hIL-17A was coated on the ELISA plate (Greiner Bio-One, Germany) for 1 h. Afterwards, the plate was washed 3× with 300 µl PBS-T/well, and the wells were blocked with PBS 1% BSA, and then incubated with 50ul of indicated antibody at a concentration range of 0 to 200 nM for 1 h. Subsequently, 50 µl of 25 nM of IL-17RA was added for 15 min and washed again three times. Secondary anti His HRP conjugated antibody (Santa Cruz Biotechnology, USA, cat: sc8036 HRP) diluted 1:250 in PBS-T was added for an incubation of 20 min at RT. Finally, the plate was washed three times with 300 μl PBS-T. The development using TMB and plate detection was performed as described for the polyspecificity assay.

### Statistics and reproducibility

ELISA assays were analyzed using GraphPad Prism 9. For competitive ELISA assay, a nonlinear fit of Inhibitor concentration vs. response—variable slope (4 parameters) was used, and for the polyspecificity a, 2-way ANOVA was used. Data presented in Figs. 3b, 5b, and Supplementary Fig. 9 are two technical replicates, and trendlines for Fig. 3b and Supplementary Fig. 9, and the mean for Fig. 5b. Functional IC50 values presented in Table 1 represent the mean ± SD on *n* = 3 individual experiments.

### HexRF—a predictor of native-like antibody-antigen interfaces

HexRF is an algorithm we developed to evaluate the potential of antibodies to bind to new epitopes, and thereby serve as templates for engineering novel specificity to the desired antigen. We used this method to evaluate the AAL160 template for engineering binding to an IL-17A epitope. The HexRF classifier was trained to identify native-like antibody-antigen docking poses generated by Hex algorithm^37^. To create such a tool, we used a non-redundant dataset of antibody-antigen crystal structures and trained a random forest classifier to discriminate between random decoys and poses that retained a high percentage of native contacts observed in the crystal structure. As input features, we used properties of antibody–antigen interface such as surface size, amino acid composition, and specific interactions, as well as the distribution of docking scores among the docked poses. Neither the AAL160 antibody structure nor its homologs were used in the training of HexRF.

### Retraining the PpRF classifier

PpRF is a random forest classifier for pairwise interactions of antibody-antigen residue positions^13^. Since its first publication, it has been retrained with an updated training set. The new training set was built from PDB structures from the SAbDab database^38^ (October 2016). We used crystal structures of antibodies with a resolution better than 3.0Å that were co-crystallized with a protein antigen. Single-chain antibodies were excluded, and redundant entries were removed. Two entries were considered redundant if their antigens share^13,27,28^ sequence identity of >95% and their Paratome CDRs (ABRs)^39^ have a sequence identity of more than 85%. Following this procedure, the new dataset had 302 PDB entries. Using cross-validation (fourfold), the retrained classifier had an Area Under ROC Curve of 0.774 for the heavy chain and 0.78 for the light chain in comparison with 0.728 and 0.778 reported on the first version of the classifier^13^. This leads to the conclusion that the performance of the classifier was mostly improved through the prediction of the heavy chain interactions.

### Aggregation prediction

Clone 114_R5_C12 was modeled using AAL160 as a template, with the homology modeling tools in the Schrödinger software. The model was analyzed using the Agg Score tool in Schrödinger, as well as with the aggregation propensity prediction tool/script from Discovery Studio, to identify residues predicted to contribute to aggregation propensity.

### Crystallization and structural analysis, AAL160 Fab

The AAL160 Fab was generated by papain digestion at 37 °C in the presence of 2.5 mM cysteine. The reaction was stopped with the papain inhibitor E64 and the Fab purified over a Protein A Sepharose Fast Flow column.

Crystals of the free AAL160 Fab were grown at room temperature by the technique of vapor diffusion in hanging drops in 24-well VDX crystallization plates (Hampton Research) by mixing 2 μl Fab (20 mg/ml in 10 mM Tris-HCl, 25 mM NaCl pH 7.4) with 1 μl of crystallization buffer (50% PEG 200, 0.1 M CHES pH 9.5) and equilibrating against the same buffer.

X-ray data were collected at 120 K with a MAR345 image plate at the Swiss-Norwegian beamline of the European Synchrotron Radiation Facility. The crystal was mounted in a cryo-loop and directly frozen in the cold nitrogen stream. In total, 246 images were collected with 1.0° oscillation each, using an exposure time of 40 sec per frame and a crystal-to-detector distance of 340 mm. The diffraction data were processed and scaled with the HKL program suite version 1.6.1^40^. The structure was determined by molecular replacement with AMoRe^41^, using the V_H_V_L_ and C_H1_C_L_ domains of PDB entry 1MIM as independent search models. The structure was initially refined with X-PLOR 98.0^42^ and O 6.2.1^43^. This structure was recently re-refined with autoBUSTER(2.11.7)^22^.

### Crystallization and Structural analysis, AAL160 Fab/IL-1β

A 1.5-fold excess of recombinant human IL-1β was added to the AAL160 Fab and the complex was purified by SEC on a HiPrep 16/60 Sephacryl S-100 column in 10 mM Tris pH 7.4, 25 mM NaCl. After concentration to 77 mg/ml, crystals of the Fab complex were grown as before in 24-well VDX crystallization plates by mixing 1.5 μl of the protein complex with 0.5 μl of crystallization buffer (2.0 M ammonium sulfate, 0.1 M Tris pH 8.5). One crystal was briefly transferred to a cryo-protectant (2.24 M ammonium sulfate, 30% glycerol, 70 mM bicine pH 9.0), mounted in a nylon CryoLoop (Hampton Research), and frozen in the cold nitrogen stream for data collection at 120 K. The diffraction data were collected with a MAR300 image plate system mounted on a FR591 Enraf-Nonius rotating anode generator. In total, 210 images were collected with 1.0° oscillation each, at a crystal-to-detector distance of 240 mm. Raw diffraction data were processed and scaled with the HKL program suite version 1.9.1^40^. The structure was determined by molecular replacement with AMoRe^41^, using PDB entry 2I1B^44^ and the V_H_V_L_ and C_H1_C_L_ domains of the free AAL160 Fab as independent search models. Initial refinement was carried out with X-PLOR 98.0^42^ and O 6.2.1^43^. This structure was utilized for the library design and all computational analyses. Final refinement was performed with autoBUSTER(2.11.7)^45^. Because of the limited resolution of the diffraction data (3.30 Å), all hydrogen atoms with null occupancy were included to maintain good model geometry. In addition, target-structure restraints were applied, based on the free AAL160 Fab structure refined at 2.00 Å resolution and an in-house, high-resolution (1.32 Å) structure of human IL-1β. Only one translation/libration/screw (TLS) group was refined per protein chain.

### Crystallization and Structural analysis, 11.003 Fab/IL-17A

The 11.003 Fab was generated by IgG cleavage on papain beads and purified over Hi-Load Superdex 26/600 GE in 10 mM HEPES, 25 mM NaCl, pH 7.0. Recombinant human IL-17A (amino acid 34–155 of Uniprot entry Q16552 with Cys129 mutated to Ser) was mixed with a 1.5-fold molar excess of the 11.003 Fab and the complex was purified by SEC (S-200 16/60) and concentrated by ultrafiltration to 16 mg/ml in 25 mM Hepes pH 7.5, 150 mM NaCl. Crystals were grown by the vapor diffusion in sitting drops technique in Innovadyne SD2 96-well plates at 20 °C, by mixing 0.2 μl of protein stock with 0.2 μl of crystallization buffer (0.2 M sodium potassium tartrate, 20% PEG 3350) and equilibrating against the same buffer. Crystals appeared after 5 days and grew slowly to full size within 2 weeks. One crystal was cryo-protected with a 1:1 mix of the reservoir solution with 30% PEG 3350, 30% glycerol and then flash-cooled into liquid nitrogen. X-ray data were collected at the Swiss Light Source (Villigen, Switzerland), beamline X10SA, with an Eiger pixel detector, using 0.999802 Å X-ray radiation. In total, 2700 images of 0.10° oscillation each were recorded at a crystal-to-detector distance of 200 mm and processed with autoPROC version 1.1.7^46^, using a resolution cutoff based on CC^1/2^ ^47^ statistics. The structure was determined by molecular replacement with Phaser^48^ using PDB entry 4hr9 (IL-17A) and the structure of the free AAL160 Fab V_H_V_L_ and C_H1_C_L_ domains as search models. The structure was refined by multiple cycles of electron-density inspection and model rebuilding in Coot 0.9^49^, followed by automated refinement with autoBUSTER (2.11.7)^45^.

### Cell-based assay for inhibition of IL-17A

The inhibition of IL-17A-induced IL-6 production was evaluated with the BJ cell line (human dermal fibroblasts). Briefly, BJ cells were seeded at a density of 5 × 10^3^ cells per well in a 96-well plate, and after an o/n incubation in culture medium at 37 °C the cells were stimulated with 1 nM recombinant human IL-17A (Novartis Basel, Switzerland) in the presence or absence of a concentration range of antibodies. After another overnight incubation at 37 °C the cell supernatants were collected, and the levels of IL-6 were determined by ELISA (BioLegend California, USA). The measured IL-6 data points were exported to EXCEL software and IC_50_ values were calculated by plotting dose-inhibition curves for the logistic curve fitting functions using EXCEL/XLfit4 software.

### Reporting summary

Further information on research design is available in the Nature Portfolio Reporting Summary linked to this article.

### Supplementary information

Supplementary Information

Description of Additional Supplementary Files

Supplementary Data 1

Reporting Summary

## Data Availability

Crystallographic atomic coordinates and structure factors have been deposited in the Protein Data Bank^10^ (www.rcsb.org) with accession codes 7Z2M: 11.003 Fab complex with IL-17A; 7Z4T: AAL160 Fab complex with IL-1b; 7Z3W: unliganded AAL160 Fab. Source data for experiments done by ELISA are available in Supplementary Data 1. All other relevant data are available from the corresponding author on request.

## Code availability

Structural biology software packages Schrodinger BioLuminate (2018; http://schrodinger.com/) and BIOVIA Discovery Studio Visualizer (https://discover.3ds.com/discovery-studio-visualizer-download) were used for visualization and aggregation prediction. The screening of the antibody database by protein-protein docking was carried out using Hex v8 (available at http://hex.loria.fr). The results were analyzed by custom scripts written in Perl and Python. The model and related scripts were deposited in Zenodo^50^.
