# Supplementary material for: “Redirecting an anti-IL-1β antibody to bind a new, unrelated and computationally predicted epitope on hIL-17A”
Source: Commun Biol. 2023 Sep 29;6:997. doi: 10.1038/s42003-023-05369-x (PMC10542344; doi:10.1038/s42003-023-05369-x)
Supplement: Supplementary file 1 — Supplementary Information [file 42003_2023_5369_MOESM1_ESM.pdf]

“Redirecting an anti-IL-1β antibody to bind a new, unrelated and computationally predicted epitope on hIL-17A”

Supplementary figures

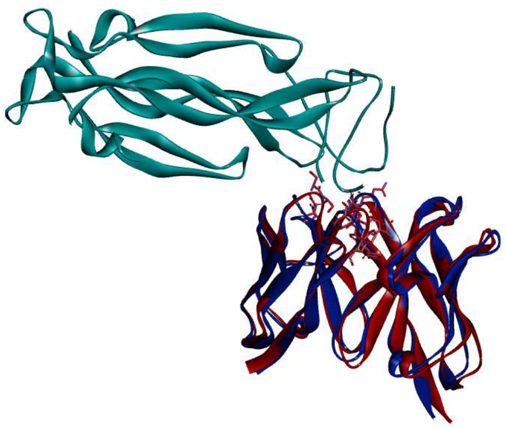

Supplementary Figure 1: Superposition of AAL160 and H142 (PDB 5N7W). AAL160 is shown in red and H142 is shown in blue. Positions that were varied in the 11.1 library are shown in stick representation.

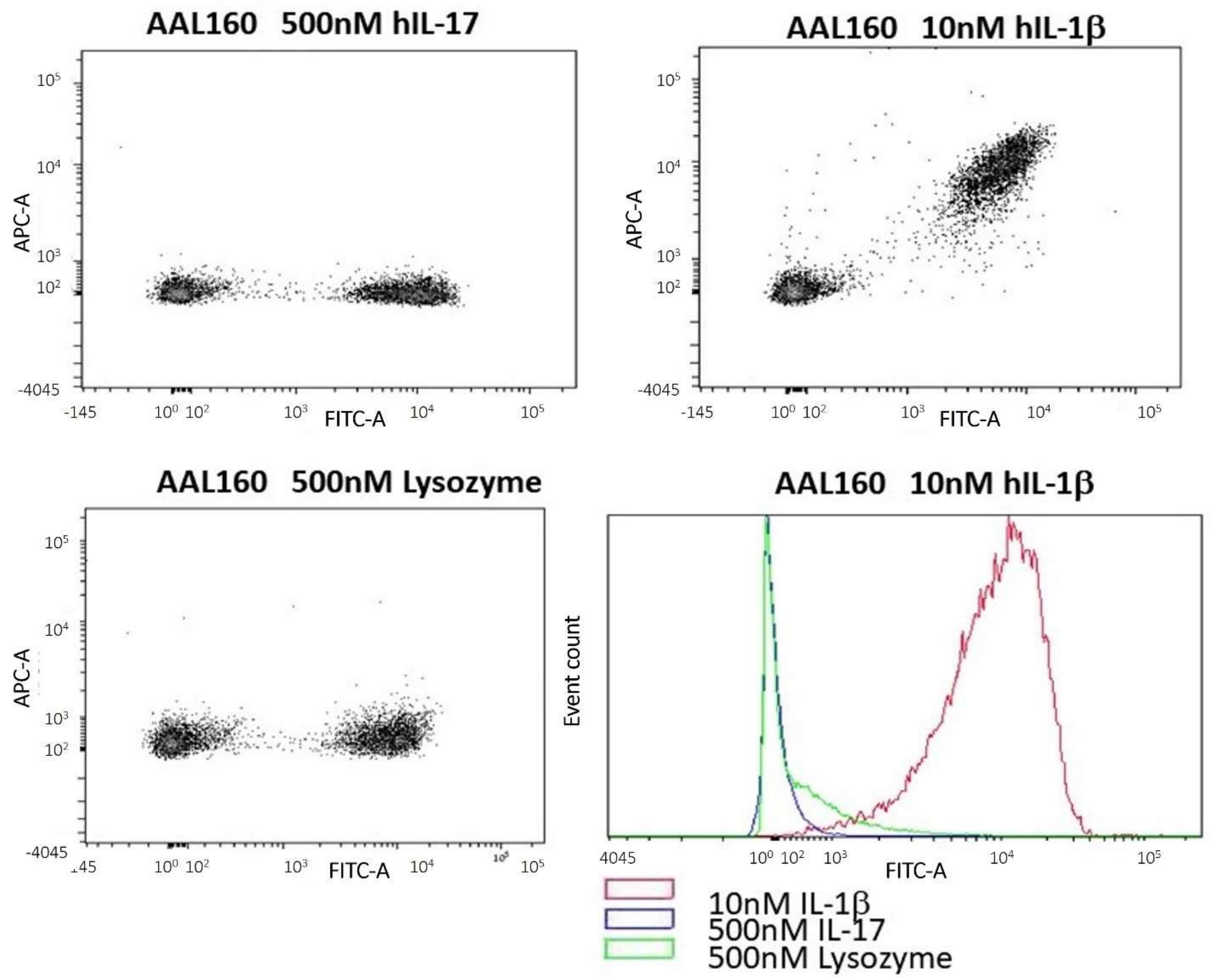

Supplementary Figure 2: Yeast surface display of AAL160 binding to IL17A and IL-1β in scFv format. Binding of AAL160 to (A) hIL-17 (500nM), (B) hIL-1β (10nM) and (C) Lysozyme (500nM). Panel D presents comparative binding to all reagents.

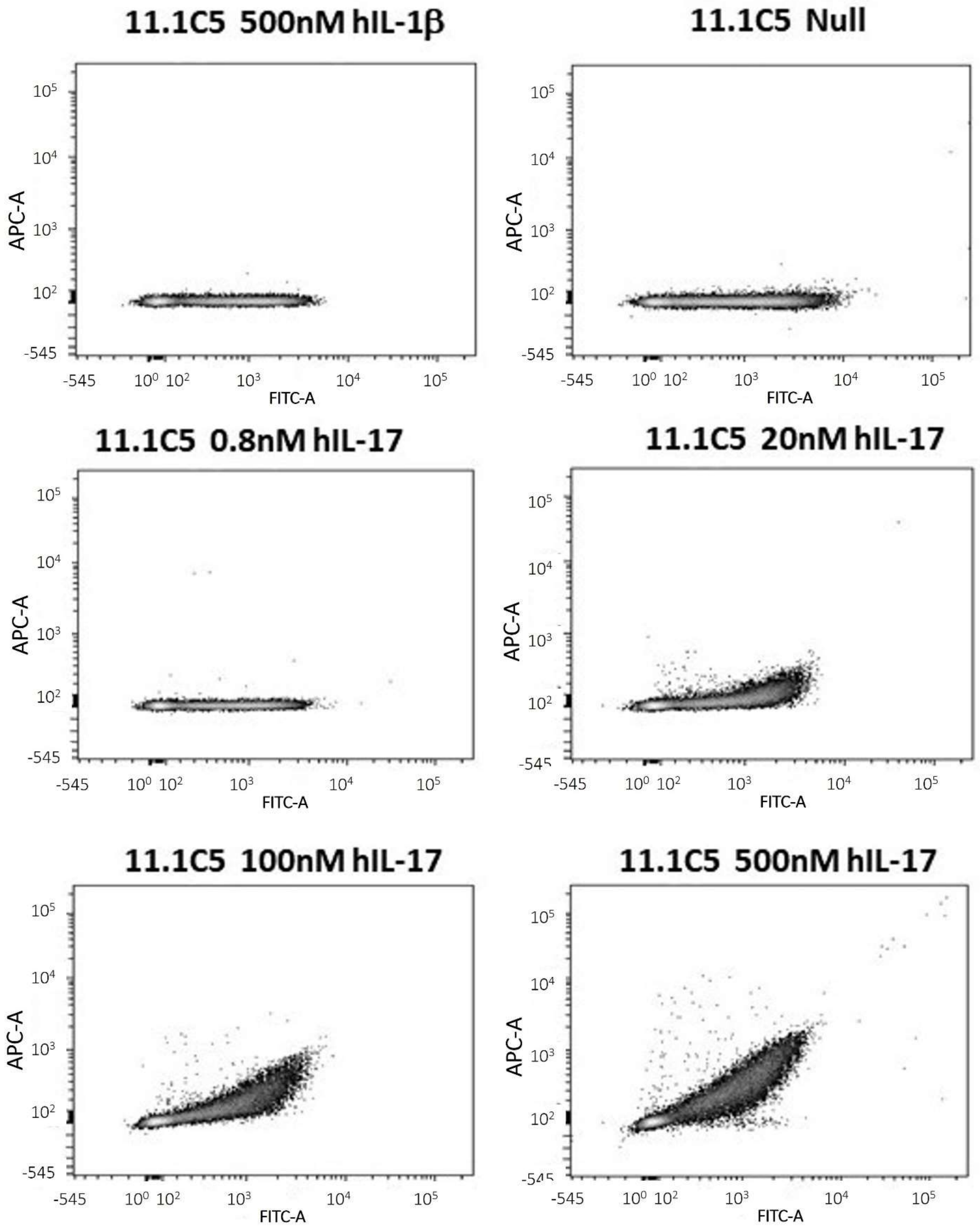

Supplementary Figure 3: Yeast clone 11.1C5 dose dependent binding to IL-17A. Yeast were incubated with the indicated concentrations of IL-17A and IL-1β.

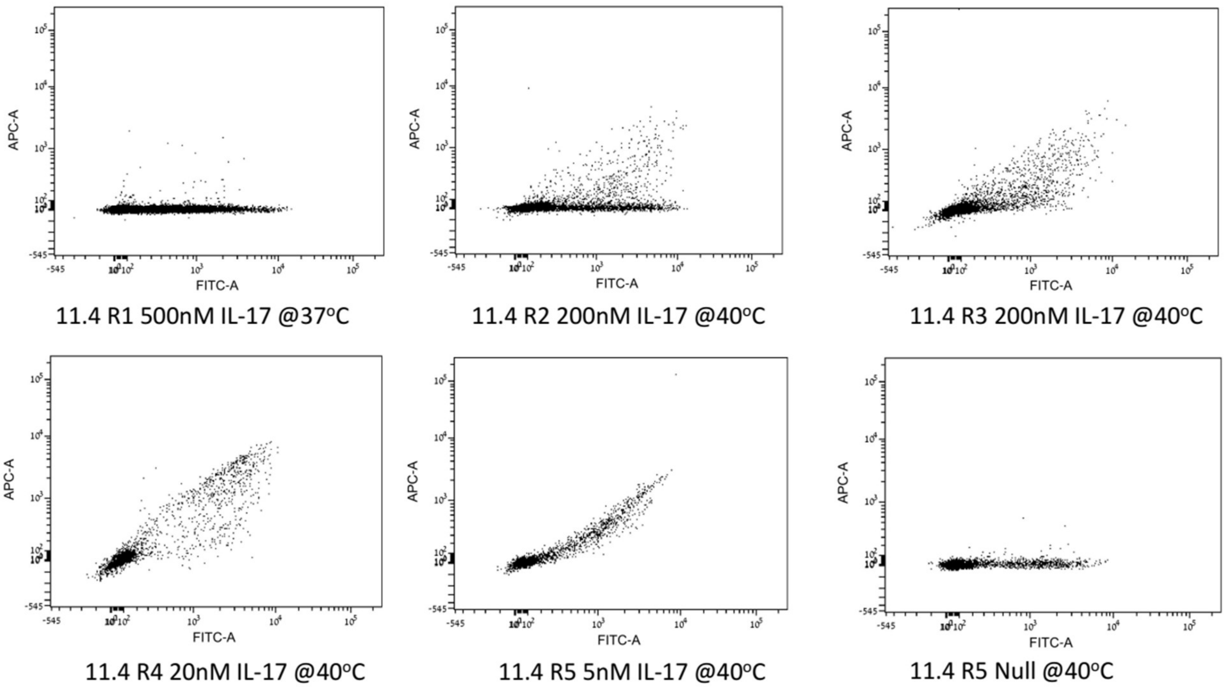

Supplementary Figure 4: Selection progression of library_C_11.4 through five rounds of sorting.

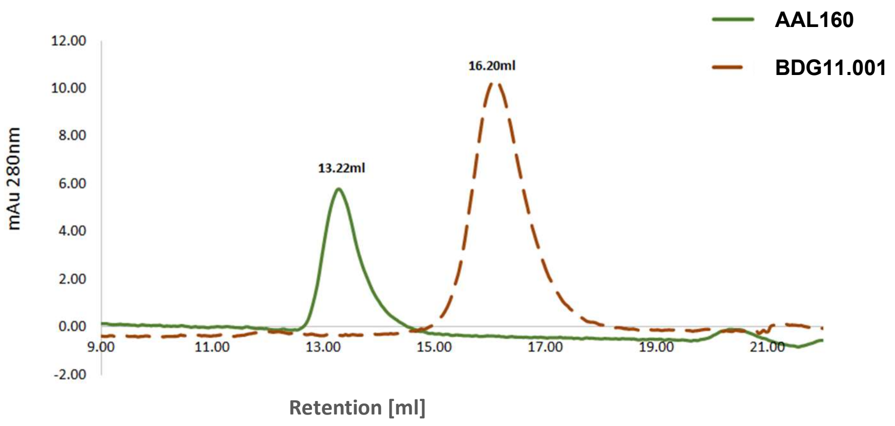

Supplementary Figure 5: SEC analysis of AAL160 and clone 11.4C_11.001 in IgG format (BDG11.001). 100ug antibody sample was run on a superdex 200 10/300 at 0.8ml/min with PBS as mobile phase (representative figure of two runs).

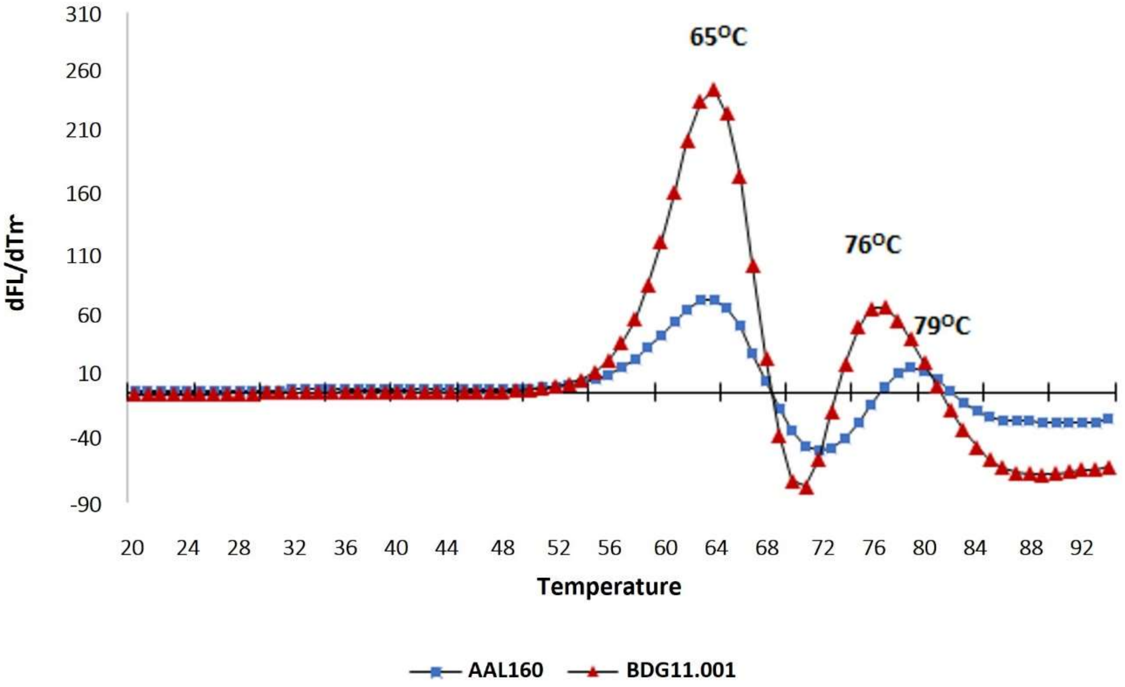

Supplementary Figure 6: DSF analysis of BDG11.001 and AAL160, inflection points of Tm1 and Tm2 are indicated (mean of 3 repeats).

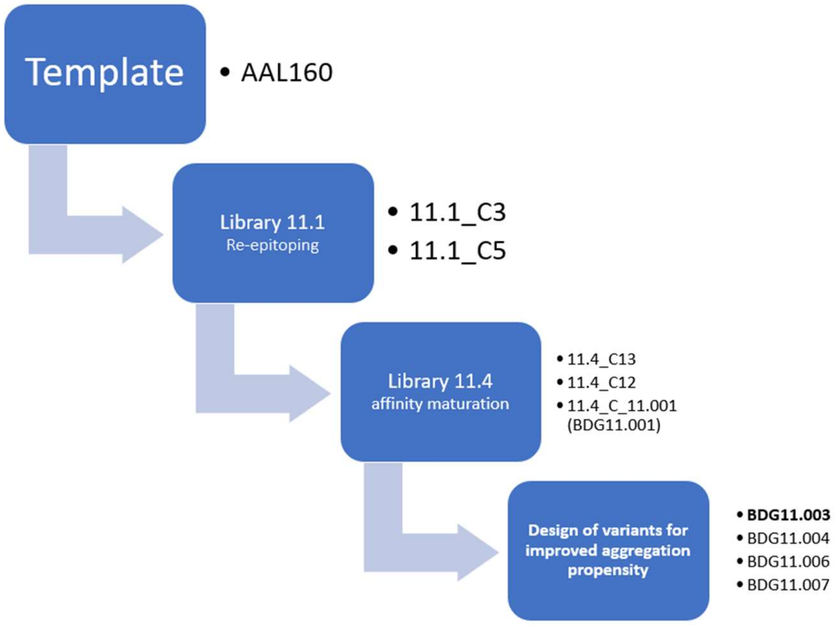

Supplementary Figure 7: workflow of the variants presented in this work.

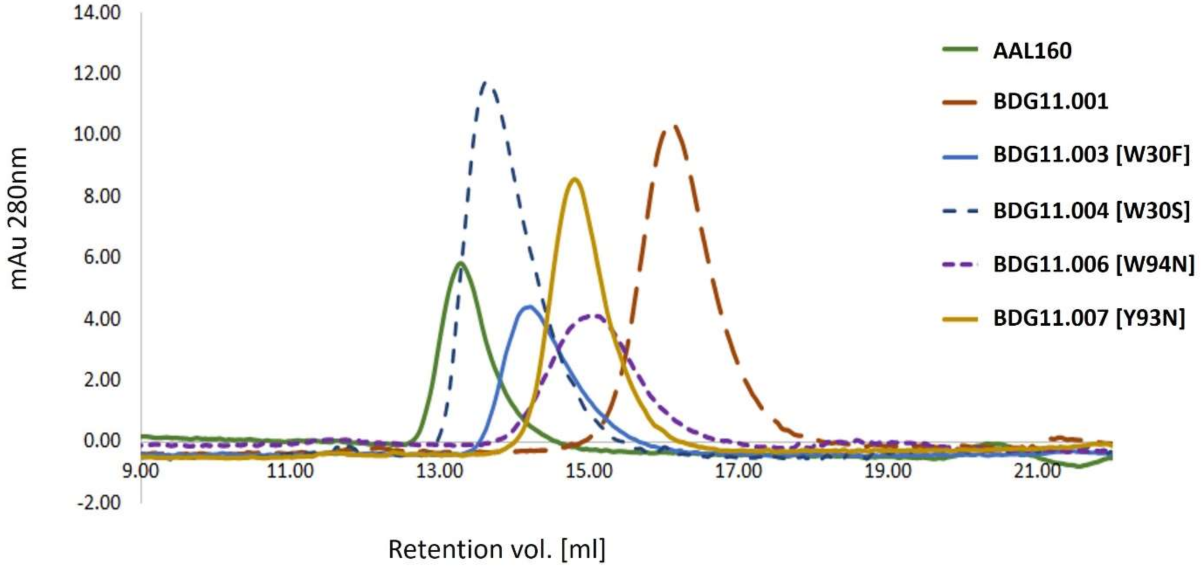

Supplementary Figure 8: SEC analysis of AAL160 and clones BDG11.001, BDG11.003, BDG11.004, BDG11.006 and BDG11.007 in IgG form. 100ug antibody sample was run

on a superdex 200 10/300 at 0.8ml/min with PBS as mobile phase (representative figure of two runs).

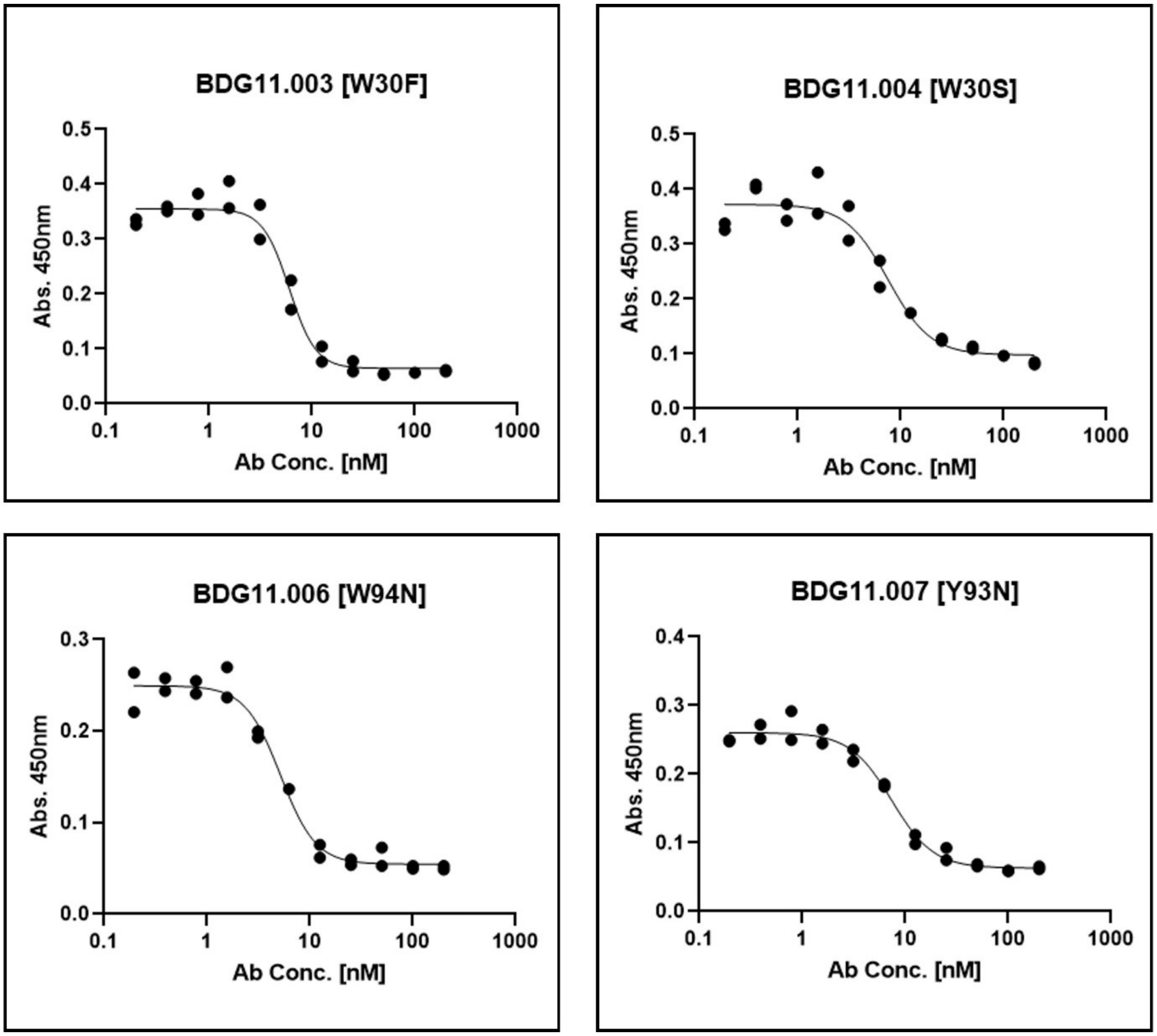

Supplementary Figure 9: BDG11.003 [W30F], BDG11.004 [W30S], BDG11.006 [W94N] and BDG11.007 [Y93N] competition with the IL-17RA receptor. Abs. 450nm signal correlates with IL17A RA binding to IL17A. Confidence intervals of IC50 for BDG11.003 [W30F] is 5.1-7.7, for BDG11.004 [W30S] is 5.7-11.1, for BDG11.006 [W94N] is 4.5-6.3 and for BDG11.007 [Y93N] is 6.1-9.7. Analysis was done in GraphPad Prism 9 using [Inhibitor] vs. response – variable slope (4 parameters). Data are n=2 technical replicates.

AAL160HC EVQLVQSGAEVKKPGESLKISCKGS**GYSFTSYW**IGWVRQMPGKGLEWMGI**IYPSDSDT**RY
IGHV551*01 EVQLVQSGAEVKKPGESLKISCKGS**GYSFTSYW**IGWVRQMPGKGLEWMGI**IYPGDSDT**RY

AAL160HC SPSFQGQVTISADKSISTAYLQWSSLKASDTAMYYC**ARYTNWDAFDIW**GQGTMVTVSS--
IGHV551*01 SPSFQGQVTISADKSISTAYLQWSSLKASDTAMYYCAR--------------------

AAL160LC EIVLTQSPATLSLSPGERATLSCRAS**QSVSSY**LAWYQQKPGQAPRLLIY**DAS**NRATGIPA
IGKV311*01 EIVLTQSPATLSLSPGERATLSCRAS**QSVSSY**LAWYQQKPGQAPRLLIY**DAS**NRATGIPA

AAL 160LC RFSGSGSGTDFTLTISSLEPEDFAVYYC**QQRSNWMFP**FGQGTKLEIK
IGKV311*01 RFSGSGSGTDFTLTISSLEPEDFAVYYCQ------------------

Supplementary Figure 10: AAL160 aligned to its closest IMGT germline sequences, CDRs are underlined.

a.

```
4NP4_H   EVQLVQSGAEVKKSGESLKISCKGSGYSFTSYWIGWVRQMPGKGLEWMGIFYPGDSSTRY
4HWB_H   EVQLVQSGAEVKKPGESLKISCKGSGYSFTSYWIGWVRQMPGKGLEWMGVIYPGDSYTRY

4NP4_H   SPSFQGQVTISADKSVNTAYLQWSSLKASDTAMYYCARRRNWGNAFDIWGQGTMVTVSS
4HWB_H   SPSFQGQVTISADKSISTAYLQWSSLKASDTAMYYCARMPNWG-SFDYWGQGTLVTVSS

4NP4_L   EIVLTQSPGTLSLSPGERATLSCRASQSVSSSYLAWYQQKPGQAPRLLIYGASSRATGIP
4HWB_L   EVVLTQSPGTLSLSPGERATLSCRASQSISSSYLAWYQQKPGQAPRLLIYGASSRATGIP

4NP4_L   DRFSGSGSGTDFTLTISRLEPEDFAVYYCQQYGSSTWTFGQGTKVEIK
4HWB_L   DRFSGSGSGTDFTLTISRLEPEDFAVYYCQQYE----TFGQGTKVEIK
```

b.

```
6APD_H   EVQLVESGGGLVKPGGSLRLSCAASGFTFSSYSMNWVRQAPGKGLEWVSSISSSSSYIYYAD
3H42_H   EVQLVESGGGLVKPGGSLRLSCAASGFTFSSYSMNWVRQAPGKGLEWVSSISSSSSYISYAD

6APD_H   SVKGRFTISRDNAKNSLYLQMNSLRAEDTAVYYCARLGYCSGGSCH-FDYWGQGTLVTVSS
3H42_H   SVKGRFTISRDNAKNSLYLQMNSLRAEDTAVYFCARDYDFWSAYYDAFDVWGQGTMVTVSS

6APD_L   -SVLTQPPSVSGAPGQRVTISCTGSSSNIGAGYDVHWYQQLPGTAPKLLIYGNSNRPSGV
3H42_L   ESVLTQPPSVSGAPGQRVTISCTGSSSNIGAGYDVHWYQQLPGTAPKLLISGNSNRPSGV

6APD_L   PDRFSGSKSGTSASLAITGLQAEDEADYYCQSYDSSLSGFYVFGTGTKVTV
3H42_L   PDRFSGSKSGTSASLAITGLQAEDEADYYCQSYDSSLSGS-VFGGGTKLTV
```

Supplementary Figure 11: Paratope residues of germline-like antibodies are conserved in pairs of aligned antibodies with high sequence identity that bind unrelated antigens. Paratope residues that are 5A from the antigen in the crystal structure are highlighted in blue, non-conserved positions are highlighted in red. **A.** Antibodies for Clostridium difficile toxin B (PDB 4NP4) and IL-13 receptor alpha (PDB 4HWB); **B.** Antibodies for RSV F protein (PDB 6APD) and PCSK9 (PDB 3H42)

Supplementary Table 1: AAL160/IL-1β and 11.003/IL-17A binding interfaces. Amount of buried solvent-accessible surface and surface complementarity were computed with the AREAIMOL and SC from the CCP4 [37] program suite. Epitope and paratope residues were identified with the CCP4 program NCONT using a distance cut-off of 5.0 Å

Complex	AAL160 / IL-1β	11.003 / IL-17A			
Interface # (chain names)	1 (LH-I)	1 (LH-GI)	2 (CD-GI)	3 (AB-JK)	4 (EF-JK)
Fab buried surface<br>• light-chain<br>• heavy-chain<br>Total Fab (Å²)	-272.8<br>-498.0<br>-770.8	-346.3<br>-528.3<br>-874.7	-564.7<br>-615.7<br>-1180.3	-323.7<br>-536.0<br>-859.7	-361.8<br>-534.0<br>-895.8
Antigen buried surface<br>• 1st subunit<br>• 2nd subunit<br>Total antigen (Å²)	-831.3<br><br>-831.3	-66.8<br>-768.3<br>-835.2	-788.5<br>-397.0<br>-1185.5	-52.0<br>-802.0<br>-854.0	-810.3<br>-54.2<br>-864.5
Total buried surface (Å²)	-1602.1	-1710	-2366	-1714	-1760
Surface complementarity	0.782	0.728	0.729	0.730	0.732
Nb of epitope residues<br>• 1st subunit<br>• 2nd subunit	22	 2<br>25	 25<br>8	 1<br>25	 25<br>1
Nb of paratope residues<br>• light-chain<br>• heavy-chain	 7<br>15	 7<br>14	 13<br>20	 8<br>14	 7<br>14

Supplementary Table 2: Residues in contact with the protein antigen in the parent and re-epitoped antibody, and corresponding germline amino-acid. A distance cut-off of 5.0Å between non-hydrogen atoms was used for the identification of contact residues. Positions marked in bold are shared by both complexes. Side-chains that differ from the germline sequence are highlighted in bold red.

CDR	AAL160/IL-1β	11.003/IL-17A	Germline residue
H-CDR1	**Ser28**	**Ser28**	Ser
	**Thr30**	**Thr30**	Thr
	**Ser31**	**Ser31**	Ser
	Tyr32		Tyr
	**Trp33**	**Trp33**	Trp
H-CDR2	**Ile50**	**Ile50**	Ile
	**Tyr52**	**Tyr52**	Tyr
	**Ser54**	**Ser54**	Gly
	**Asp55**	**Asp55**	Asp
	**Asp57**	**Asp57**	Asp
	**Arg59**	**Arg59**	Arg
Outer loop		Lys74	Lys
H-CDR3	**Tyr99**	**Tyr99**	
	Thr100		
	**Asn101**	**Asn101**	
		Tyr102	
	Asp103		
N-terminus		Glu1	Glu
		Ile2	Ile
L-CDR1		Gln27	Gln
	**Ser30**	**Phe30**	Ser
	Ser31		
	**Tyr32**	**Tyr32**	Tyr
L-CDR2			
L-CDR3	Arg91		Arg
		**Phe92**	Ser
	**Asn93**	**Tyr93**	Asn
	Trp94		Trp
	**Phe96**		Pro
