# Supplementary material for: “Redirecting an anti-IL-1β antibody to bind a new, unrelated and computationally predicted epitope on hIL-17A”
Source: Commun Biol. 2023 Sep 29;6:997. doi: 10.1038/s42003-023-05369-x (PMC10542344; doi:10.1038/s42003-023-05369-x)
Supplement: Supplementary file 2 — Description of Additional Supplementary Files [file 42003_2023_5369_MOESM2_ESM.pdf]

Description of Additional Supplementary Files

File name: Supplementary Data 1
Description: Measurements for ELISA assays for Figure 3b, Figure 5b, Supplementary Figure 9, Supplementary Figure 10.
